# Enhancing equity within the promotions dossier evaluation—Strategies to mitigate biases

**DOI:** 10.3389/fpubh.2025.1697387

**Published:** 2026-01-09

**Authors:** Tisha R. Joy

**Affiliations:** Department of Medicine, Schulich School of Medicine & Dentistry, Western University, London, ON, Canada

**Keywords:** academic promotion, physician, equity, teaching evaluation, publication, letter of reference, award, candidate statement

## Abstract

Gender, racial, and ethnic disparities in career advancement in academic medicine continue to exist despite ongoing calls to diversify the physician workforce and leadership representation. Compared with white men, women and racially and ethnically underrepresented physicians are more likely to enter academic medicine, but less likely to achieve promotion. Cited barriers to academic advancement include ambiguous or inconsistent criteria for consideration for promotion and lack of standard processes for reviewing applications. The promotions committee therefore is an important rate-limiting step subject to biases that impact not just tenure, but also leadership opportunities and physician salaries. This perspective article explores areas where biases influence promotion criteria evaluation, including but not limited to accomplishments within teaching, research, and/or service, external recognition, candidate statements, and reference letters. The article then will propose strategies helpful to promotion committee members to help mitigate these biases and provide more equitable promotion evaluation.

## 1 Introduction

Fostering a diverse academic physician workforce is a critical step in addressing health inequities (1). Physicians underrepresented in medicine (URM; including those who are racialized, have disabilities, are gender and/or sexually diverse) are more likely to contribute to scholarship, education, and provision of services and programs for communities aligned with their identities (2–4). Greater patient satisfaction, increased adherence to preventative services, and improved health outcomes have also been demonstrated when physician-patient concordance exists (5–9).

Growth of faculty from underrepresented races has been steady but slow, with 31% of U.S. medical school faculty being racialized (10, 11). Efforts to increase medical student diversity has not yet translated into faculty diversity, partly due to waning interest for academic medicine (12, 13). One identified barrier related to interest in academic medicine was lack of clarity concerning promotion and tenure. Promotion is key for tenure and career advancement, including access to leadership and potentially financial opportunities. The increased need for faculty to meet clinical demands balanced with a growing desire for improved work-life integration prompted several changes related to promotion and tenure, such as incorporation of part-time tenure tracks, extension of tenure probationary periods, and institution of tenure clock-stopping policies (e.g., for parental leave) (14–16). Despite these efforts, diversity for academic physicians is highest at entry-level ranks and decreases thereafter (17, 18). Compared with white men, physicians who are women and/or racially diverse are more likely to enter academic medicine, but less likely to achieve promotion (18). In addition to gender and racial/ethnic disparities, career track disparities also occur in promotion. Clinician educators are less likely to achieve career rank compared to researchers (19).

Dissatisfaction with career progress and serious intent to leave commonly occur in clinical faculty, and the lack of appropriate recognition within promotions evaluations was one of the strongest predictors for intent to leave, demonstrating the importance of equitable promotion practices for faculty morale and retention (20). Clinical faculty are evaluated for promotion based on their contributions in education, research and service (clinical care/administration/leadership). Promotion criteria vary from institution to institution. However, inconsistent application of promotion criteria and lack of awareness of biases inherent to several metrics used for evaluation may unintentionally impact promotion acceptability within any given institution. Mallon and Cox (16) found that implicit bias education for promotion committee members was commonly offered, but not necessarily mandatory. Importantly, implicit bias education typically centers on an individual's beliefs and not necessarily systemic biases occurring within the methods used for evaluation. Similarly, since promotions dossier evaluations may occur over many months, efforts for bias awareness and mitigation at an individual level may decrease over time.

This perspective article therefore explores areas of bias potentially embedded within the evaluation of promotion dossier content such as accomplishments within teaching, research, and service; external recognition; candidate statements; and reference letters. Although the article will at times provide specific considerations for clinical faculty, the strategies proposed for mitigation are applicable for the equitable evaluation of all faculty.

### 1.1 Evaluating the components of the promotion dossier

Teaching and research are core pillars of all higher education institutions. For clinical faculty, specific career tracks such as Clinician Educator, Clinician Researcher, Clinician Scientist exist based on differential weighting of education and/or research deliverables. In the past, although over 70% of faculty identified teaching as their main interest, most felt that academic recognition systems favored published research, prompting changes to the definition of scholarship and the development of an education portfolio (EP) as methods to value and structure research and educational contributions for promotions evaluation (21–23). Although the majority of academic physicians are clinician educators, recent data still show that they are less likely to achieve promotion compared to those with a research focus, despite these changes (19, 24).

#### 1.1.1 Teaching contributions

The EP or teaching dossier captures five main contributions: (1) teaching (any activity that fosters learning such as courses or instructional material development), (2) assessment (learner evaluations), (3) curriculum (innovations), (4) mentorship and advising, and (5) leadership (committee or other work to drive educational change) (22). In addition, a statement of teaching philosophy outlining personal teaching beliefs and approach to teaching is often included. Despite adoption of the EP as a streamlined method to evaluate teaching, early studies revealed that learner evaluations of educators still remained the primary evidence utilized within promotion committees (25, 26). Similarly, given variabilities in length and content as well as uncertain EP evaluation guidelines for promotion committees, the utility of EPs remains low and has prompted efforts for redesign (26–29).

Regardless of these approaches, there are inherent biases in how teaching is evaluated for clinical faculty. Firstly, the majority of teaching by clinical faculty occurs not in classrooms or courses, but rather in an apprenticeship model in inpatient and/or outpatient clinical settings, where learners are often scheduled with multiple faculty over a set time period. Classroom or course-based teaching primarily occurs only in undergraduate medical education, and even then, depending on institution, the majority of this teaching occurs in the early years and may be shared amongst a number of faculty members due to their patient care commitments. In other words, often multiple clinical faculty teach within a single course/block, and some faculty may only conduct a single large or small group session as part of that course. Learner evaluations are often not effectively captured in these settings. Evaluations are instead typically distributed at the end of a themed session (e.g., diabetes block), undergraduate course (e.g., Clinical Pharmacology), and/or clinical rotation (e.g., General Surgery). In efforts to avoid evaluation fatigue, learners are not routinely required to provide evaluations on all faculty they have encountered, but rather are offered the option to provide feedback on the course or clinical rotation itself. Sometimes, learners may choose to evaluate certain faculty members based on recency bias (i.e., faculty members with whom the learners had most recently worked) and/or due to a particularly positive/negative experience with a faculty member. Interestingly, students' perception of likability accounted for two-thirds of the total variance of evaluations of a faculty member even without knowing how the class was taught (30). Given this understanding, faculty with lower likeability and those who do infrequent and/or short teaching engagements will be less likely to receive evaluations compared to those with higher likeability and/or those providing longer-term consistent exposure. Thus, evaluations may not be equitably obtained for all clinical faculty engaged in teaching due to this distribution system.

Secondly, learner evaluations have been shown to be influenced by student interest in the topic, level (introductory vs. higher), and faculty demographics and therefore do not necessarily reflect teaching effectiveness (31). Although there is variability in published studies, reviews have demonstrated that learner evaluations can reflect underlying biases regarding gender, race, ethnicity, and/or sexual identity of their instructors (31, 32). Women, racialized individuals, those with accents, those with Asian last names, and those with intersectional identities (e.g., women of color) receive lower learner ratings (31, 32). In an online teaching environment where the gender of the instructor was manipulated, learners provided lower evaluations if they believed the instructor was a woman, despite identical course delivery (33). Abusive comments have been increasingly noted, primarily directed at URM faculty (32). This feeds into concerns by faculty that evaluators including promotion committees do not know what normal variation in ratings/comments is acceptable and therefore may focus excessively on rare negative ratings/comments, with implications for promotion and tenure as well as the wellbeing and mental health of faculty (34, 35). The influence of faculty gender on learner evaluations has been demonstrated for academic clinicians as well (36, 37). Although more data specifically examining the relation between teaching evaluations and sexual identity, race, ethnicity for clinical faculty are needed, it is plausible that these biases may also exist to varying degrees, similar to non-clinical faculty. Thus, reliance on learner evaluations as evidence of teaching effectiveness may impact URM clinical faculty promotion acceptability, if promotion committees remain unattuned to possibilities of bias.

Thirdly, the definition of scholarship for education in most institutions continues to focus on traditional metrics such as peer-reviewed publications and/or teaching of learners *within* the faculty member's home institution. These metrics however may not always be most suitable for clinical faculty, especially clinician educators. The scholarship of discovery (building knowledge through research) is ideally disseminated through peer-reviewed publication (21). In order to reflect the diversity of faculty interests, Boyer (21) included three other forms of scholarship: integration (interpreting the use of knowledge across disciplines; i.e., interdisciplinary work), application (helping to address societal issues), and teaching (studying models and practices for learning). The concept of broadening scholarship to 4 distinct yet overlapping areas was well-received, but how quality of scholarship would be determined was uncertain. Glassick (22) therefore identified 6 standards for assessing scholarship–evidence of clear goals, adequate preparation, appropriate methods, significant results, effective presentation, and reflective critique. Importantly, to be considered scholarship, the work should also be publicly available, available for peer review/critique, and able to be built upon or reproduced (22). In this digital age of easy access to health (mis)information online, provision of learning and educational materials to learners as well as to the public at large is important. Digital and social media, podcasts, blogs, online open-access sites and new apps are effective methods for mass reach, and may be more likely to be used by clinician educators. However, despite the changing landscape for education dissemination and frameworks for how to evaluate social media as a metric for inclusion in promotions, only 8% of allopathic medical schools in the United States accepted digital and social media output as evidence of scholarship for promotion consideration (38–40). Thus, although changes in the definition of scholarship were widely appealing, the majority of institutions and by extension, promotion committees have not innovated to include modern metrics that could impact the promotion potential of faculty, especially clinician educators. As the majority of clinician educator track physicians are female, the lack of modernization of scholarly output definitions may differentially affect women (41). Ultimately, without careful attention to these biases related to inequitable evaluation distribution for clinical faculty, learner bias within learner evaluations of faculty, and lack of inclusion of more modern methods of scholarship output, promotion committees may inadvertently perpetuate the disparities in promotion seen for URM faculty.

#### 1.1.2 Research contributions

Clinical faculty may participate in varying types of research, such as basic science, translational, clinical, quality improvement, patient safety, educational, community participatory and epidemiologic. These can be further classified as investigator-initiated vs. industry-sponsored or industry-supported, based on who has designed and/or funded the project. Research contributions tend to focus on quantifiable metrics related to scholarly output, grants, and mentoring and advising. Scholarly output metrics often include number of presentations and publications, impact factor of the journals published in, and indices related to impact on the research community, such as h-index. Greater recognition within promotions often occurs for greater number of publications, senior/corresponding or first-author status in journal publications, publication in journals with higher impact factors, higher h-indices, principal-investigator status for grants and research studies, investigator-initiated research, and national and international conference presentations (42). Since research brings in monetary reward as well as reputation to the faculty member's institution, greater recognition tends to be for researchers who have higher grant funding amounts and/or have received grants from funding organizations with national/international reputation (e.g., National Institutes of Health).

Goodhart's law states “When a measure becomes a target, it no longer becomes a good measure.” Utilizing target metrics such as the quantitative research metrics listed above may cause individuals to adjust their behaviors in order to meet them, potentially instigating adaptive negative behaviors focused on manipulating the system. Despite their longstanding use, research-related metrics are subject to Goodhart's law, and thereby have hidden effects on promotions committee evaluations of URM faculty in particular. Firstly, the h-index, which is an author-related metric measuring both productivity and citation impact, is influenced by time, self-citation, and name changes, with the latter primarily affecting women and transgender individuals. Since a higher number of citations will positively impact the h-index, both self-citation and coercive citation influence the h-index. Importantly, men have been found to self-cite 70% more often than women (43). Gendered effects are not merely related to self-citation. In promotion and tenure deliberations, below-average h-indices were more harshly judged for URM faculty than for non-URM faculty, with amplified effects occurring in the context of intersectionality, particularly for racialized women (44). Coercive citation, whereby editors of journals “request” the authors of a manuscript under review to include citations to other articles within their journal, impacts not only the h-index of the relevant authors cited but also the journal's impact factor (45, 46). The practice of honorary authorship (the inclusion of authors who did not contribute to the research but who often hold either influence or power) increases the h-index of the honorary author(s) (46). Researchers with longer duration of scientific careers may have higher h-indices due to increased number of citations over time. Thus, individuals who may have taken leaves, whether parental, pregnancy, and/or medical-related, may have shorter “scientific age” compared to individuals who have not taken leave, despite similar appointment starts. Thus, when promotions committees evaluate dossiers, the duration of “active” presence within academia, excluding the leave period, needs to be taken into account.

Secondly, journal impact factors are not a useful measure of a researcher's impact although are often considered in deliberation of promotions, grants, and/or awards. Several have advocated for impact factors not to be used at all for these purposes (47, 48). Importantly, a journal's impact factor can be influenced by coercive and self-citation, and is not representative of the research impact of all individual articles within that journal (45–47). Instead, impact factors are often driven by a smaller number of highly cited papers overshadowing the rest. In fact, Ogden and Bartley (49) published a list of methods for editors to increase a journal's impact factor, including advice to publish potentially high-citation manuscripts earlier in the year so they have a longer time period for citations. Longer articles and review articles tend to be heavily cited and increase the impact factor of journals, resulting in preferential publishing, while more niche-related and/or non-traditional research may lack access to journals with high impact factors (47). Thus, researchers with niche or non-traditional research fields may have not only less opportunities to publish in higher impact factor journals but also be inadvertently penalized by promotion committees for doing so. Similarly, journal impact factors can be specifically influenced by societal trends. For example, journals with COVID-19-related publications showed a significant increase in their impact factors (50). Thus, changes in journal impact factor by external factors and/or specific manipulation make it a biased metric for researcher impact. Promotions committee members therefore should avoid journal impact factor from unduly influencing evaluations of researcher scholarly output.

Thirdly, grant success as a measure of researcher academic success has several limitations. Honorary authorship was evident in clinical grants with mentors and/or investigators with reputation often being added in efforts to increase grant success (46). It could be posited that this approach would be particularly helpful for grant success from more prestigious funding agencies, where the investigator with reputation may have networking capital. The practice of honorary authorship thereby perpetuates the academic and granting success of already established investigators. Conversely, faculty who have less networking, are early career, and/or conduct research in very specific niche areas may be less likely honorary authors and less likely to garner funding from highly prestigious national/international organizations. Ginther et al. (51) demonstrated that grants submitted to the National Institutes of Health (NIH) by African American/Black applicants were less likely to be funded compared to those by white applicants, and only 25% of the funding disparity was due to traditional research output metrics (52). Although white women were just as likely to receive an NIH grant as white men, racialized women were less likely than white women to do so, pointing to the influence of intersectionality on grant success (53). Grant funding success not only directly impacts the ability of a faculty member to perform their research and publish, but has downstream effects related to the ability to recruit graduate/post-doctoral students who in turn would contribute further publications and thereby increase the research output metrics relevant to promotion committees. Lack of grant funding for URM faculty may therefore have amplified effects as well as impacts on salary as research investigators with grants may sometimes receive salary support through the grant (54). Interestingly, despite the emphasis placed on grant funding, research impact has been demonstrated to be only weakly linked to funding (55). Impact per dollar was lower for large-grant holders, and researchers who held funding from multiple granting sources did not have greater impact than researchers who held a single national grant, challenging the hypothesis that larger grants lead to greater discoveries (55).

Fourthly, publications and author by-line position may affect evaluation of URM faculty. Ross et al. (56) demonstrated that women in research teams are significantly less likely than men to be credited with authorship and/or recognized in the development of a patent, supporting the Matilda effect. The listing of co-first authors is increasingly common (57, 58). In clinical articles where co-first authors were of differing genders, a lower proportion of female authors were listed first (57). Thus, despite efforts to recognize contributions equally, the tendency of promotion committees to place emphasis on either first or senior author position may adversely impact women who are co-first authors due to the more common placement of women as second author instead (57). Ethnic disparities in authorship have also been evident in medical journals, and non-native English speakers may face additional pressures and barriers to publication and/or presentation (59, 60). Certain fields of medicine such as genomics may be impacted by growing author lists, due to the need for large international research collaborations. Hyperauthorship may influence citation counts, while simultaneously impacting the ability to distinguish individual contributions within the publications and allowing for honorary authorship to go unnoticed (61). Meanwhile, although interdisciplinary and collaborative work is increasingly recognized within medical schools, this type of research also does not fare well for allowing unique contributions of individual faculty members to be valued within promotions evaluations, as traditional criteria (such as author position and/or principal investigator status) tend to be emphasized (62). Locally conducted research and/or certain fields of research may in addition influence publication acceptability. For example, researchers who conduct quality improvement, patient safety and/or educational research impacting practice and/or policy particularly at a local/institutional level may have difficulty finding appropriate journals for publication or national/international conferences for presentation. Thus, including policy and practice changes (even without publication) as equivalent to peer-reviewed publications would be helpful to ensuring equity in promotions evaluations for researchers conducting these types of research.

#### 1.1.3 Service contributions

Service can be characterized according to typically two main categories–internal (activities directly related to the faculty member's division, department, and/or institution) and external (activities outside of one's institution such as to the profession and/or regional, national, and/or international “community”). Some forms of service are compensated (e.g., department chair) while other forms are often uncompensated (e.g., mentorship). Compensated service roles such as department chair may be viewed as evidence of leadership and administration abilities and thereby given weight within promotions evaluations, while uncompensated service activities such as mentorship, advising, peer/learner support, and community education may be acknowledged but often may also carry less weight compared to research and/or teaching activities (63). URM faculty tend to perform valuable yet often uncompensated service, and the term “minority tax” or diversity tax has been used to describe the extra responsibilities placed on URM faculty to advance diversity initiatives at the institutional level, with resultant adverse effects on time available for promotion- and tenure-valued activities such as research and teaching (64). When controlling for rank, race/ethnicity, and field of department, women faculty have been shown to perform significantly more service than men (63). Racialized women felt additional obligations to volunteer their time for service due to their race (65). Despite advocacy for fostering a mentorship program within many institutions as a method to advance research and recruitment, mentoring is not met with as much recognition as “direct” research- and/or education-related activities within promotion and tenure evaluation (66). Importantly, for compensated service roles such as department chairs, women continue to be underrepresented, even among female-dominated specialties such as obstetrics and gynecology (67). Thus, URM faculty not only do more uncompensated service which takes time away from promotion and tenure-valued activities, they are less likely to have compensated roles like department chair which may be also more favorably valued in promotions evaluations, resulting in a “double whammy.”

#### 1.1.4 Other components of the dossier—External recognition, candidate statement(s) and letters of reference

Awards and speaker invitations, particularly nationally and/or internationally, often provide additional evidence of an individual faculty member's recognition in the academic community, and are valued by promotions committees. Research-related awards may rely on publication number, journal impact factor, h-index and/or citation counts, and grant success, while education-related awards may rely on learner evaluations, publications, and perhaps peer/learner narrative comments. However, the inherent issues with these metrics for URM faculty as noted above may impact recognition of URM faculty. In fact, women and racialized individuals have been noted to receive national awards and awards associated with speaker invitations at lower rates compared to non-URM faculty (68–70). Mentorship awards are increasingly common, and yet not as easily recognized within promotion and tenure policies (66). Importantly, URM physicians are also underrepresented as invited speakers at medical conferences (71, 72). Thus, although awards and speaker invitations may be beneficial as “added evidence” of promotional acceptability, they may not be equitably distributed as they rely on active networking, sponsorship, as well as the use of fair adjudication methods.

Narrative statements by the candidate include the candidate statement (outlining their main contributions and rationale for being considered for promotion) as well as teaching philosophy if applicable (outlining personal teaching beliefs and approach to teaching). Other narrative components of the promotion dossier are the referee letters, often written by internal/external peers, learners, and rarely, other members (e.g., hospital leaders) able to speak to the faculty member's contributions to research, teaching and/or service. Little is written about the importance of candidate-written statements to evaluation decisions or about the metrics used. Candidates have been advised to ensure proper grammar, punctuation, and spelling as well as an articulate style to make the application more readable which may help with reviewer “confidence” in the evidence presented (73). Thus, the ability to write a convincing and articulate narrative may be important, but the aspect of self-promotion has received less overt attention. Self-promotion does not come easily to all individuals. For example, women who served as first and senior authors of research articles were less likely to use positive terms, such as *novel, unique*, or *promising* to describe their own findings and instead were more likely to use words such as *supportive*, thereby downplaying their results; this differential in self-promoting words was associated with fewer citations of their work (74). Thus, even when scientifically relevant data is presented, self-promotion language may play a role in “convincing” others about the importance of that data. Whether URM physicians self-promote differently on candidate-written documents warrants further appraisal. Meanwhile, external reference letters have been given emphasis as referees are meant to be neutral when evaluating the faculty member's contributions and impact within academia based on the institution's promotion guidelines. Concerns exist related to gender-related differences in the frequency of agentic vs. communal words, references of status, doubt-raising statements, as well as in the length of the reference letter (75, 76). Madera et al. (77) demonstrated that linguistic features (e.g., clout, authenticity) in external reference letters were linked to writer characteristics (e.g., the writer's h-index) rather than the faculty member's performance indicators, thereby calling into question the neutrality of these letters in promotion decisions. As previously noted, within promotion deliberations below-average h-indices were more harshly judged for URM faculty than for non-URM faculty, with amplified effects occurring for racialized women (44). Interestingly, when external referees highlighted URM faculty scholarship within their letters, the differential evaluation of URM faculty was mitigated, indicating that reference letters can sway promotions committee evaluations to even mitigate biases (44). Thus, tips to writing helpful reference letters including methods to mitigate bias have been proposed by Gottlieb et al. (78).

## 2 Discussion

**Figure 1:**
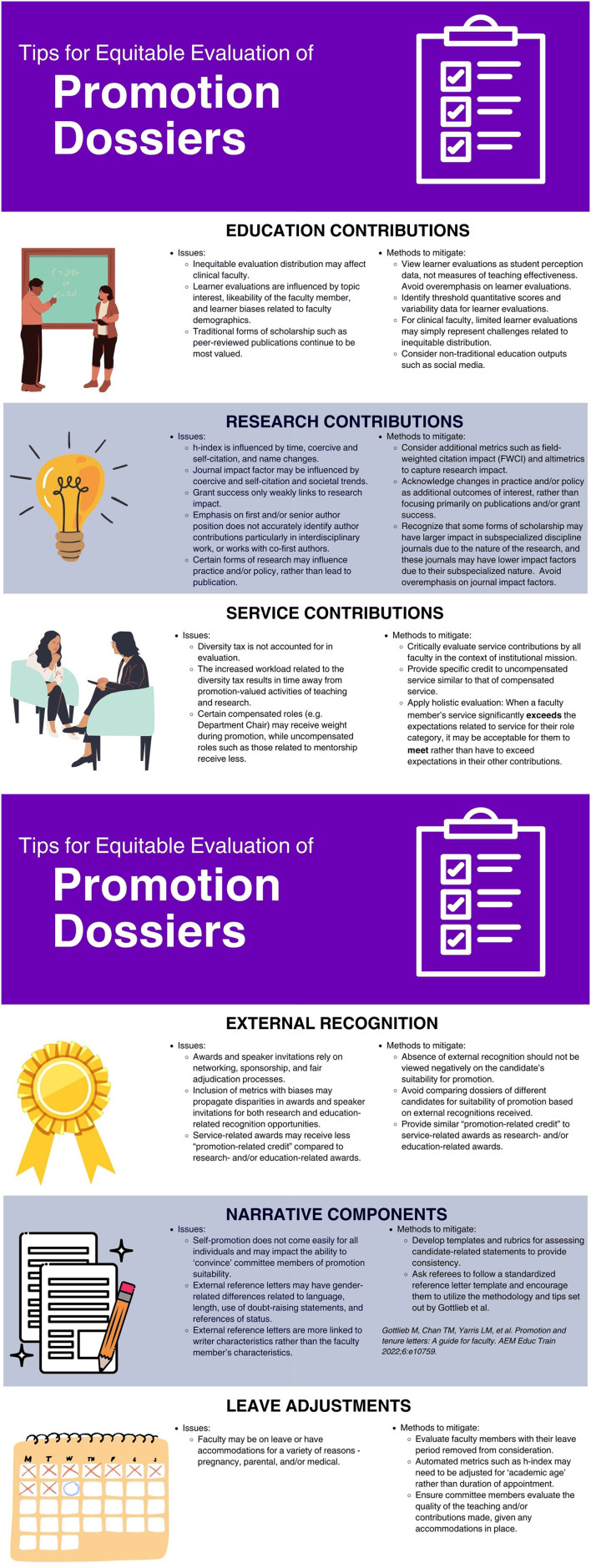
An infographic tool for equitable promotions dossier evaluation.

Promotions committee members can address the above noted structural biases in a few critical ways (Figure 1). Evaluation of teaching by promotion committees typically relies on learner evaluations and traditional methods of scholarship. To mitigate these biases, promotion committee members need to first recognize that learner evaluations are not direct measures of teaching effectiveness but rather merely represent student perception data (79). Keeping this in mind, promotion committees should avoid undue emphasis on quantitative scores and/or comparison of quantitative scores between faculty members especially since learner evaluations may be impacted by personal interest/motivation, course level, and faculty demographics. Instead, thresholds of acceptability should be determined in advance, against which all dossiers are evaluated. For example, the committee might identify that a score of 5 on a Likert scale of 1–7 is acceptable, and unless a faculty member consistently receives scores below 5, their teaching is perceived to meet an acceptable standard. Similarly, excessive focus by committee members on a few negative comments or a few scores that do not meet the threshold should also be avoided, given the biases that may occur based on instructor demographics. Instead, from a quality improvement lens, institutions and committee members could examine the previous year's data related to teaching evaluations to identify percentages for negative comments and/or negative scores (i.e., scores that did not meet the minimum threshold established) to determine “normal” variability. This could help identify and mitigate significant changes in negative comments/scores that may occur over time. Committee members should also recognize that for clinical faculty, the absence of learner evaluations for content delivery may merely reflect differences in the method of teaching delivery by clinical faculty compared to non-clinical faculty (i.e., most of clinical teaching occurs not in courses/classrooms where evaluations are more likely to be distributed, and those with limited classroom sessions may not receive evaluations regardless). In general, an overemphasis on learner evaluations for determining promotion acceptability for clinical faculty should be avoided.

While the majority of universities continue to prioritize traditional metrics for scholarship for teaching (e.g., peer-reviewed publications), promotion committee members should reflect on how they can still value modern scholarship such as education through social media even within the confines of their institutional metrics, rather than being dismissive, especially as this helps to foster inclusivity through reward and recognition of faculty. Given that the lack of appropriate recognition within promotions evaluations was one of the strongest predictors for intent to leave, utilizing metrics published and those already implemented at other institutions for inclusion of social media within promotion allows promotion committees to potentially impact faculty retention (20, 38–40).

For research evaluations, promotion committee members need to avoid explicitly and/or implicitly considering impact factor of journals, citation counts, grant funds, and the h-index in determining researcher impact and promotion acceptability. The San Francisco Declaration on Research Assessment (SF-DORA), the Leiden Manifesto, and the Hong Kong principles have all advocated for the move away from currently used publication and citation metrics to find improved and more equitable measures of research impact (48, 80, 81). Valuing research that demonstrates impacts on policy and/or practice has also been advocated for, which is especially impactful for clinicians who may work in educational, quality improvement, patient safety, and/or community participatory research since policy and practice, rather than publications, may be their primary output (48). Moher et al. (81) outlined five principles for evaluating research output: responsible research practice; transparent reporting; open science; valuing diverse types of research; and recognizing all contributions to research and scholarly activity. Some newer metrics meant to mitigate the biases with traditional metrics include field-weighted citation impact (FWCI) and altimetrics (https://altimetric.com) (82). For example, FWCI is meant to reflect the performance of a paper within the research field, rather than the journal within which it was published, by comparing the citations of the paper to the average citations expected in the field. Similarly, altimetrics aims to capture the impact of research beyond citation count, such as policy documents, social media effects, thereby allowing researchers to demonstrate a more inclusive evaluation of their research not just in the research community but also in the public. Unfortunately, FWCI may also still be influenced by highly cited outlier publications, similar to journal impact factor (82). Thus, while numerical metrics may seem easily implementable, no single metric seems without influence/bias, and Goodhart's law needs to be heeded. Ultimately, research impact should be assessed based on scientific content rather than publication metrics (48). Gagliardi et al. (83) identified five measures deemed to be of high importance to measure research quality and impact: (1) research advances existing knowledge; (2) research plan is innovative; (3) an independent body of research (or fundamental role) is supported by peer-reviewed research funding; (4) research outputs are relevant to discipline; (5) quality of the content is evident in dissemination. Promotion committees should therefore consider including acknowledgment of changes in practice and/or policy as an acceptable outcome and utilizing additional metrics of FWCI and altimetrics for research impact to foster equitable evaluation for all researchers, particularly those in niche fields, non-traditional methods, educational scholarship, and quality improvement or patient safety. Similarly, it is important to recognize that given challenges faced by researchers in these fields, they may not have access to journals with higher impact factors, and thus some forms of scholarship may have a larger impact in a specific discipline journal with lower impact factor. Encouraging structured letters of reference by peers (external) to outline the five measures noted by Gagliardi et al. (83) may also be of greater assistance in helping promotion committees evaluate scientific impact especially as Masters-Waage et al. (44) also demonstrated the influence letters may have in mitigating biases during promotions deliberations.

Mentoring/advising remains a key service role amongst most institutions to help faculty and learners navigate the academic environment and provide an inclusive place to thrive; however, the value of these efforts in promoting the “diversity” profile of the institution are not engrained in most promotion and tenure evaluations. Similarly, to mitigate the effects of the diversity tax, promotion committees should evaluate how service activities link to the institutional mission, and value uncompensated service just as equally as compensated service. Committees should likely examine the impact of service contributions internally and/or externally that far exceed the expectations of the member's role category and recognize that these may have been tasked to these particular members due to their identities, inadvertently placing them at risk from achieving “exceeds expectations” in other typically valued activities. Committees can mitigate biased evaluation by fostering a holistic promotion review wherein a faculty member who exceeds in service may still be promotion acceptable if they “meet expectations” in the areas of teaching and/or research.

Since awards and speaker invitations have demonstrated gender and racial/ethnicity biases, the absence of these external recognitions should not be considered a negative by promotions committees; instead these should be seen as “nice to have” criteria. Similarly, comparison of dossiers of faculty members based on external recognitions should be avoided in determining thresholds for acceptability, by promotion committees. The impact of narrative statements on promotion evaluations are subject to bias and may be influenced by choice of language, length, readability, and self-promotion. It would be ideal if promotion committees could develop standardized reference letter templates and encourage referees to use the methodology outlined by Gottlieb et al. (78). And finally, given that faculty may take leave and/or have accommodations for a variety of reasons, it is critical that promotion committees evaluate the faculty member based on their contributions with the leave period removed. Specifically, promotion committees will need to evaluate research-related (including automated metrics like the h-index) and teaching-related contributions based on “academic age” rather than from time of appointment. Also, if accommodations were made (decreased clinical and/or teaching load), committee members should focus on the quality of the teaching and/or curricular contributions accomplished, not necessarily the quantity.

Diversifying the physician workforce has been viewed as a way to help address health inequities; and yet, specific disparities related to promotion and retention are evident for URM faculty within academic centers. Promotion committees hold significant power in determining access to tenure as well as potentially financial and leadership opportunities. Implicit bias education focused on individual biases is important and committee members should actively remind each other of these over the course of the promotion cycle. Yet, attention should also be given to biases inherent to the metrics used for evaluation of the promotion dossier. Many of the strategies presented in this article are highly feasible with limited cost (e.g., setting thresholds of acceptability for teaching evaluations, incorporating FWCI and/or altimetrics, reformatting reference letter guidelines, assessing for bias in reference letters), while some require more concerted shifts in mindset (e.g., valuing service-based recognition similarly to education- or research-based recognition, recognizing limitations of grant success/amounts and/or journal impact factors), and some would require institutional level support (e.g., adoption of DORA guidelines, incorporation of social/digital media as a form of scholarship within promotion criteria). However, what is required is time, effort, and dedication by individuals who will innovate and advance promotion evaluations to be modernized for today's faculty. Furthermore, systemic and institutional level changes still need to occur to ensure promotion committee diversity, regular policy updating, and fair committee processes (voting, number of reviewers per dossier), as these were beyond the scope of this perspective. The attached infographic (Figure 1) can serve as a visual reference tool for committee members of the issues and mitigation strategies for traditional metrics when evaluating. Ultimately, even within an institution's current practices, several of the strategies provided can be implemented now as a step toward facilitating equitable recognition and reward for all faculty.

## Data Availability

The original contributions presented in the study are included in the article. Further inquiries can be directed to the corresponding article.
